# Minimally Invasive Treatment of Urolithiasis in Children: Evaluation of the Use of Flexible Ureterorenoscopy and Laser Lithotripsy

**Published:** 2020-05-31

**Authors:** A. Garzi, M. Prestipino, E. Calabrò, R.M. Di Crescenzo, M.S. Rubino

**Affiliations:** 1 Division of Pediatric M.I.S. and Robotic Surgery University of Salerno, Italy; 2 Division of Pediatric Surgery A.O. S. Maria della Misericordia Perugia, Italy; 3 Department of Advanced Biomedical Sciences, Pathology Unit, University of Naples Federico II

**Keywords:** urolithiasis, pediatric age, flexible ureterorenoscopy, laser lithotripsy

## Abstract

Urolithiasis is a multifactorial disease; in recent years, its incidence has gradually increased in pediatric age. Among the factors involved in urolithiasis pathophysiology, urinary tract anomalies and metabolic diseases are the most relevant, although ethnicity and environmental factors may have an important role.

The advances in technology and miniaturization of endoscopic devices have permitted the use of Retrograde Intrarenal Surgery (RIRS) to treat kidney and ureteral stones.

Nowadays, flexible ureterorenoscopy and laser lithotripsy, which are techniques that have been applied in the management of adult upper urinary tract disorders, are also used in children as a minimally invasive treatment of urolithiasis with encouraging, effective and safe results.

The Authors report a retrospective review of their record of cases considering 21 pediatric urolithiasis treatment procedures performed between October 2017 and April 2019 in a total of 17 patients (10 males and 7 females).

Six procedures involved the use of the flexible ureterorenoscope (FURS) while in 15 procedures the application of the laser fiber was used (FURSL). A case of laser lithotripsy for bladder stone was included. The average age of patients was 10.5 years (2–18 years). The renal pelvis dilatation pretreatment was evaluated in post-operative follow-up.

From the evaluation of the sample in analysis, the use of RIRS has good results in the treatment of paediatric urolithiasis, emerging as a valid option in the management of the paediatric population in terms of efficacy and safety, with an improvement in patient outcomes.

## I. INTRODUCTION

The incidence of paediatric urolithiasis has increased in the recent years (1, 2). Many factors contribute to the onset of this disease: developmental anomalies of the genito-urinary system, metabolic abnormalities and geographical location have effects on the formation of stones (3). The innovations in surgical techniques and a better knowledge of the pathology allowed the evolution of the treatment of urinary tract stones.

Open surgery is no longer the first choice technique for the treatment of upper urinary tract stones in both adults and children; over time it has given way to shock-wave lithotripsy and endourological techniques such as Semirigid Ureterorenoscopy (URS) and Retrograde Intrarenal Surgery (RIRS) which drastically changed the option of treatment. Since their first use in the 1980s, there has been a continuous improvement in instrumentation and a greater propensity to use flexible ureteroscopes, with the possibility to combine the application of the Holmium laser to fragment the identified stones.

Success rates were demonstrated for the treatment of renal stones with flexible ureterorenoscopy (FURS) in children, comparable to those observed in adults. FURS has minimal morbidity and by providing a direct visualization, it allows the treatment of multiple stones in different positions within the kidney and ureter (4, 5).

FURS and flexible ureterorenoscopy and laser lithotripsy (FURSL) were shown to be safe and minimally invasive procedures that can ensure a higher percentage of success rates in the elimination of stones compared to Extracorporeal Shock Wave Lithotripsy (ESWL), in particular with stones greater than one centimeter (6).

## II. AIMS

Although previous studies have investigated the use of uroendoscopic mininvasive techniques for the treatment of urolithiasis in paediatric age, their safety and effectiveness have not yet been demonstrated (7).

The aim of this study was to evaluate the performance of RIRS with FURS and FURSL procedures in terms of outcome of the paediatric patient, reduction of hospitalization time and the number of procedures necessary to ensure the remediation of the upper urinary tract; moreover the aim was to evaluate the reduction in size or the elimination of one or more stones, in case of multiple stones in a single session.

## III. MATERIALS AND METHODS

A total of 17 patients (10 males and 7 females) aged between 2 and 18 years (mean 10.5 years) with a suggestive history of upper urinary tract lithiasis were considered; a case of bladder lithiasis was also included. Patients with either urinary tract abnormalities or metabolic abnormalities or both were evaluated.

The ultrasound (US) and contrast-free CT were used as pre-operative imaging techniques to define the location and size of the stones, whereas in two cases a pretreatment uro-MRI was performed. RIRS with FURS and FURSL techniques for ureteral and renal stones was indicated.

Each procedure was performed under general anesthesia and guided by fluoroscopy.

21 procedures were carried out in the period between October 2017 and April 2019 at the Pediatric Surgery Division of the Pediatric Hospital “Istituto G. Gaslini” of Genoa and the University of Salerno. Of these, FURS was performed in six cases, FURSL in fiftheen. We considered patients per treatment group. In patients with suspected urinary lithiasis, an adequate metabolic evaluation is performed in order to identify the factors contributing to the onset of calculosis (8).

Clinical data, initial and follow-up evaluation were compared; the size of the FURSL pre- and post-treatment stones were evaluated in order to consider the response to treatment and the effectiveness of the technique.

The follow-up program included postoperative clinical and US evaluations done at 7 and 14 days.

In patients who had renal pelvic dilation in the initial evaluation, the measurement of renal pelvis in follow up was compared.

## IV. DATA SYNTHESIS AND ANALYSIS

Categorical variables were expressed as number and percentage; continuous data were reported as mean and standard deviations. Changes in renal pelvis diameter from baseline to post-operative period were assessed by using the Wilcoxon test; a p value <0.05 was considered statistically significant.

### Notes on surgical technique

The technique used is the following: Cystoscopy is performed for the identification of ureteral meatus; a guide wire is positioned on guide x-ray, thus the introduction of ureteral working channel is performed. If a pre-operative stent is already present, it should be removed first.

Therefore, the flexible ureteroscope is introduced and, going up along the ureter, the upper, middle and lower caliceal groups and the renal pelvis are explored.

Once is identified, the stone is subdivided into several fragments with laser fiber (Holmium laser) and/or the extraction of the same with “basket” or “alligator” is performed. This step is followed by the washing of the caliceal groups and the placement of the ureteral stent JJ. Laying of a bladder catheter at the end of the procedure.

The stents are usually removed after 2 weeks.

## V. RESULTS

In our experience, minimally invasive treatment of urolithiasis in children using RIRS proved to be an effective procedure in the elimination of stones and laser lithotripsy was performed in 71% of the total procedures. The main localization of the stones was at the lower calyceal group (50% of the total procedures).

The FURSL patient group included 6 males and 6 females with an average age of 10.4 years (7.6 for males and 13.7 for females). The procedure was performed in 47% of cases on the right, 40% of cases on the left while 2 procedures were limited to the laser fragmentation of a large bladder stone (13%).

The FURS patient group included 4 males and 2 females with an average age of 10 years (11.3 years for males and 7.4 years for females); among this group the procedure was performed in 17% of cases on the right, in 66% of cases on the left and bilaterally in 17%.

In the FURSL group, we observed 33% of patients with metabolic disorders including: 1 case of distal tubular renal acidosis, 1 case of hypercystinuria, 2 cases of hypercalciuria. No metabolic disorders were found in the FURS group.

Clinically, the most frequent symptom was pain, observed in 67% (FURSL) and 50% (FURS) of patients; furthermore, urinary tract infections were detected in 58% (FURSL) and 33% (FURS) of cases.

Considering FURSL group, in 7 patients were found abnormalities in the urinary tract (Table 1).

Moreover, urinary tract abnormalities in the FURS group were identified in 67% of patients (details in Table 1).

Overall, 53% of cases presented familiarity for urolithiasis.

The main localization of calcific concretions was at the level of the lower and middle calices, respectively in 58% and 13% in FURSL group, whereas the lower calyceal group was involved in 30% of the FURS patients.

Renal pelvis appeared to be affected in 4% in FURSL group and 10% in FURS group. The ureteral stones were mainly located in the distal ureter (Table 2–3). Table 3 summarize the average size of stones pre and post-treatment.

A difference between pretreatment and post-treatment was observed, with an average renal pelvis dilation of 17,2±7,1 mm pretreatment and 14,7 ±7,4 mm in follow up in FURSL; 20,3 ±8,9 mm pretreatment and 11,5 ±3,5 mm post-treatment in FURS. Considering both FURSL and FURS patients with renal pelvis dilatation, pretreatment changes in post operative period were statistically significant at p<0.05 (p-value= 0.0083).

The complete reclamation of the upper urinary tract with a single treatment was achieved in 47% of patients (40% FURSL; 33% FURS). In the remaining cases, even though we did not obtain the complete elimination of the stones by a single procedure, the rate of improvement of the patient’s condition is 33% with a reduction in the size of the stones and with an increase of the possibilities of spontaneous elimination, while reoperation with laser lithotripsy is programmed for 20% of cases.

The mean patient hospitalization was 2,3± 1,2 days for the patient FURS group and 2,9 ± 1,4 days for the FURSL. The average surgical times for the FURSL was 56 ± 25 minutes and 49 ±24 minutes for the FURS.

During FURS procedure, in two cases the stones identified in the preoperative imaging examination were not detected due to probable spontaneous expulsion. All procedures were completed without intraoperative complications.

## VII. DISCUSSION

Thanks to the update of the material and improvements in auxiliary equipment technology, the application of flexible ureteroscopy has become increasingly common in the diagnosis and treatment of stones in the upper urinary tract in children. Since flexible ureteroscopy allows lithotripsy and removal of the stones through a natural channel of the human body, surgical trauma, bleeding, kidney damage and other complications are significantly reduced compared to PCNL, Laparoscopic surgery and open surgery and the technique has an acceptable reproducibility (9).

Although in this study we considered a small population of patients and a limited number of procedures was performed, good results were obtained both in terms of elimination/reduction of the size of the stones and improvement of the outcome of the patients.

These observations support the use of endoscopic treatment, in particular with laser fragmentation; this technique, in terms of presence or absence of calculations in the post-operative period, promotes the elimination or reduction of the number and size of kidney and ureteral stones.

With regard to the patient outcome: in patients undergoing RIRS treatment, the observable reduction in renal pelvis size after treatment is one of the advantages of minimally invasive treatment of urolithiasis in children, promoting a fast return to non-pathological dilation values of the renal pelvis. This result supports the use of RIRS.

## VIII. CONCLUSION

The minimally invasive treatment with flexible ureterorenoscopy and lithotripsy, is a widely-used and repeatable therapeutic approach for treatment of urolithiasis, which favours the natural expulsion of fragmented stones, reduces patients’ morbidity, and allows a faster postoperative recovery with shorter hospitalization time.

This study, although limited by the relatively small sample size and the observational study design, suggests that the RIRS technique with the FURS and FURSL methods may be a safe and effective option for treatment of urolithiasis in paediatric age.

## Figures and Tables

**Table 1:** Urinary tract anomalies in FURSL and FURS

FURSL group	FURS group
Horseshoe kidney	Hydronephrosis(2 cases)
Nephrocalcinosis	Double renal district with hydronephrosis(1 case)
Double renal district with hydronephrosis	Neurological bladder(1 case)
Vescicoureteral reflux	
Ureteropelvic junction stenosis (UPJ)	
Bilateral congenital megaureter	
Hydronephrosis	

**Table 2:** Average size of stones - FURSL group

Stones localization	Number of stones pre(%)	Average size of stones pretreatment (mm) ± SD	Number of stones post(%)	Average size of stones post-treatment (mm) ± SD
Renal Pelvis	1 (4%)	25	1 (17%)	9
Lower calyx	14 (58%)	7,8 ± 3,5	3 (50%)	6,3 ± 1,2
Medium calyx	3 (13%)	7,3 ± 3,8	2 (33%)	8 ± 2,8
Distal ureter	5 (21%)	11,3	/	/
Bladder	1 (4%)	175	/	/

**Total**	**24**	**15,6±34,2**	**6**	**7,5 ±1,9**

**Table 3:** Average size of stones – FURS group.

Stones localization	Number of stonepre (%)	Average size of stones pretreatment (mm) ± SD	Number of stone post (%)	Average size of stones post-treatment (mm) ± SD
Renal pelvis	1 (10%)	5	/	/
Lower calyx	3 (30%)	5,4± 3,1	1 (50%)	9
Distal ureter	6 (60%)	9,8 ± 4,2	1 (50%)	15

**Total**	**10**	**8.1 ± 4,1**	**2**	**12± 4,2**
